# Comparison of Auditory Brain Stem Responses and Otoacoustic Emission of Autism with Healthy Children

**DOI:** 10.31661/gmj.v9i0.1937

**Published:** 2020-12-22

**Authors:** Seyed Gholamreza Noorazar, Yalda Jabbari Moghaddam, Rasul Kharzaee, Mojtaba Sohrabpour

**Affiliations:** ^1^Psychiatry Department of Tabriz University of Medical Sciences, Tabriz, Iran; ^2^Otolaryngology Department of Tabriz University of Medical Sciences, Tabriz, Iran; ^3^Tabriz Azad University of Medical Sciences, Tabriz, Iran; ^4^Noncommunicable Diseases Research Center, Fasa University of Medical Sciences, Fasa, Iran

**Keywords:** Autism, Auditory Brain Stem Response, Otoacoustic Emission

## Abstract

**Background:**

Autism is a neurodevelopment disorder, including difficulty in establishing relationships and social interaction, difficulty in communication, performing restricted, and repetitive behaviors. The impaired reception and integration of sensory information especially auditory data are one of the main characteristics of children with autism. According to various studies, the brain stem plays a key role in the reception and integration of auditory and sensory data. Hence, this study aims to comparison auditory brain stem responses (ABR) and otoacoustic emission (OAE) of autism patients with healthy children.

**Materials and Methods:**

This case-control study was performed on 20 autism children (4-8 years old) as case group who referred to psychiatry clinics affiliated with Tabriz University of Medical Sciences and 20 healthy age-matched as the control group. The severity of autism was evaluated by the Gilliam Autism Rating Scale (GARS). Also, ABR and OAE were recorded, and all data compared with the healthy children.

**Results:**

The latencies between the waves III-V and I-V bilaterally, and wave V bilaterally and wave I in the left ear showed a significant increase in children with autism compared to the healthy group.

**Conclusion:**

This study shows that there was a reduced nerve conduction velocity in the auditory pathway of the brain stem in children with autism compared to healthy children.

## Introduction

Autism is a neurodevelopmental disorder that causes several symptoms, including problems in establishing relationships and social interaction, difficulty in communication, and restricted and repetitive patterns of behavior [1]. According to the World Health Organization and the American Psychological Association, Autism is classified as a subgroup of pervasive developmental disorders that is caused by central nervous system disorder [1]. According to the American Psychological Association, autism disorder is defined as an abnormal or impaired development in social interaction and communication and obviously restricted pattern in person’s interests and activities [2,3]. Parents of such children are often the first to recognize impairment in the social interaction of their children [4]. It appears that autism is a genetic disorder that is caused by an impairment in several genes [5]. Because of individual differences, the severity, and the spectrum of disabilities, autism is ranged from mild to severe [6]. Autism disorder can be seen in all social and economic levels and all racial and ethnic groups [7]. The symptoms usually manifest between 18 to 36 months of age [8]. Each year, the number of children with autism increases, and based on the studies, the prevalence of autism in children reported 5 per 10,000 persons [9]. One of the noticeable features of children with autism is impairment in reception and integration of auditory data. Studies have shown that people with autism compared to healthy subjects may have impaired brain stem functions. Also, the afferent pathways can affect their efferent hearing pathways [10,11]. Various imaging, embryology, genetic, and neurobiological studies have shown brain stem involvement in these children [12-17]. According to these findings, involvement in different levels of the brain stem can affect auditory sensory input and cause difficulties in understanding and integrating auditory information at a higher level, which result in autism symptoms [18,19]. This is, to some extent, confirmed by evidence like delay in speech learning and language development along with abnormal responses to sensory input. This sensory disruption has been proved using cognitive potentials [20]. Also, an abnormal increase in ABR waves latency and abnormal increase in wave I latency has been reported due to cross olivocochlear bandle (COCB) involvement in children with autism with normal hearing, Also ABR waves changes reported in some other chronic pediatric disease [21-27]. However, some researchers believe that there is no significant difference in the latencies of waves between autistic and normal groups. Some even believe that there is a significant reduction in latency in autistic children [28,29]. Khalfa *et al*. [30] in a study using otoacoustic emission (OAE), reported a decreased inhibitory effect of COCB in children with autism. In addition, Lemaire *et al*. suggested that COCB involvement in patients with tinnitus can influence the latency of wave I [31]. The aim of this study was to the comparison of auditory brain stem responses (ABR) and OAE of autism patients with healthy children.

## Materials and Methods

###  Patients

 This case-control study was performed on 20 autism children using convenience sampling as case group who referred to psychiatry clinics affiliated with Tabriz University of Medical Sciences and 20 healthy age-matched as the control group who were randomly selected from children referred to ear, throat, and nose (ENT) specialists with nasal fracture. The diagnosis of autism was provided based on DSMIV-IR criteria.

###  Inclusion and Exclusion Criteria

 Inclusion criteria were cooperation in conducting hearing tests and ages 4-10 years. Also, children with conductive hearing disorder (perforation of the eardrum and the external ear canal atresia, etc.) were excluded from the study.

###  Data Collection

The two groups were controlled regarding speech and language disorders caused by impaired hearing. Also, the severity of autism in children was performed individually using a set of specific tests such as the Gilliam Autism Rating Scale (GARS) [32] by experienced psychiatrists and psychologists. The GARS questioner has three sections (42 questions), including stereotyped behaviors, communication, and interaction. Each section consists of 14 questions, and each question is scored from 0 to 3 points based on the repetition of behavior over a period of 6 hours. In general, patients with less than 52 points have the potential of mild autism, and those with 53-84 points have the potential for moderate autism; and those with more than 85 points have the potential for severe autism. Before performing the hearing test, the patients underwent an otolaryngology examination by ENT specialist. Also, tympanometry was performed by an audiologist, and in the absence of the conductive hearing loss, they were tested for ABR and OAE. Briefly, stimulants with expansionary polarity used to test ABR (Standard Click 125 microseconds), which produced by stimulator device 2250 ERA and presented through headphones to the ear. The intensity was 70 dB peSPL. The number of stimulus was 20 excitations in a second and 10 milliseconds intervals. Click stimulus presented to the experimental ear and responses were recorded. Band-pass filtering of the device was adjusted at frequencies 150 to 2500 Hz. The active and the reference electrodes were placed at Fz zone and A1 or A2 zone (based on the international system), respectively. The earth electrode was placed over mastoid on the opposite side of the tested ear, and electrode resistance was less than 5 kilo-ohms. Children were placed in a lying position during the test. In the case of normal results of ABR with the click stimulus and obtaining normal result in OAE, hearing loss in patients ruled out.

###  Ethical Issue

 The consent written form was obtained from the parents of the children. All individuals’ information, including their name were kept confidential, and all the steps of this study were in accordance with the Declaration of Helsinki. Also, the research project was approved by the Ethics Committee of Tabriz Azad University of Medical Sciences (IR.IAU.TABRIZ.REC.1397.0.31).

###  Statistical Analysis

 ABR waves variability among autism patients’ groups, and respected controls were illustrated using descriptive statistics (mean, range, standard deviation, standard error). Data analyzed in SPSS software (version 23.0, IBM Corporation, Armonk, NY, USA). The P-value<0.05 was used to indicate a statistically significant difference between groups.

## Results

 The mean age of autism and control groups was 7.7±1.45 and 7.8±1.9 years (ranged 4-10 years), respectively (Table-1). All ABR and OAE results in the control group were normal. Regarding autism severity, 5 children have mild, 8 children moderate, and 7 children have severe disorder. In all of the mild and moderate autism children, we found ABR waves, but in sever form; no ABR waves were observed (P<0.001). To comparison of ABR waves latency time’s results, at first, we exclude patients who had any ABR waves (6 ABR), so remnant ABR waves latency compared. The III-V and I-V interweave latency time were longer in the autism group (P<0.001). Latency time of wave V and wave I were longer in patients group (P<0.001, Table-2). In the autism group, 11 children had a positive OAE test, and 9 children had a negative OAE test.

## Discussion

According to our results, increase latency wave I was found in the left ear of mild autism children with compared to healthy children. Considering this result, it seems that impairments in the auditory processing pathway are mostly in the upper region of the brain stem of mild autism. By comparing the results of two autism subgroups with the normal group, it has been found that the waves III-V and V latency in both ears in the subgroup of severe autism was significantly higher compared to the normal group and the subgroup of mild autism. It was suggested that increasing waves’ latency in patients with severe autism reflects the involvement of lower regions (level) of the brain stem. In accordance with our result, Tas *et al*. [26] reported increased latency between waves IPL III-V and hearing impairment in children with autism. *Hamidi et al*. also showed that there was a significant difference in the average latency of waves III, I-III, V, and I-V between normal and autism groups [33]. However, the finding regarding an increase in latency of wave III did not approve the latency between the waves I-III [33]. Rosenhall *et al*. [22] reported that the latency between interpeak latency IPL (III-V) in children with autism was longer than the control group. They also showed that the latency of waves I and V in ABR was also significantly longer in the autism group [22]. Nevertheless, there has been found no evidence regarding the extended latency of wave I in the right ear. This finding was in line with the results of our study that confirmed the extended interval between the waves III-V and V in both ears as well as wave I in the left ear. Melda *et al*. reported that the notification of lack of orientation over the own name of infants at the age of 14 months may be a sign of autism spectrum disorder [34]. Doaa *et al*. demonstrated the binaural interaction component, which manifests binaural interaction to be significantly reduced in the autism patients [35]. So, there is some evidence that auditory dysfunction is a common feature of autism spectrum disorder, we have some limitations in case finding and audiologic evaluation so we recommended multicenteral evaluation etiology of this finding by the other otologists..

## Conclusion

 Although hearing screening routinely was done on all children, precise evaluation of the auditory tract of the brain stem in autism patients seemed to be necessary to early diagnosis and more prevention of patients’ disability.

## Acknowledgment

 Authors thankful to EMA24 English editing service to improve the language of this article.

## Conflict of Interest

 The authors declare that they have no conflict of interest.

**Table 1:** Demographic Information

**Variables**	**Case group** **(n=20)**	**Control group** **(n=20)**
**Age, y ( mean±SD )**	7.7±1.45	7.8±1.9
**Gender, n( %)**		
Male	12(60)	11(55)
Female	8(40)	9(45)

**Table 2:** ABR Wave in Autism Patients

**ABR waves**	**Groups**	**Number**	**Mean**	**SD**	**P-value**
**I-R**	Autism	14	1.5	0.12	0.99
	Normal	20	1.49	0.13	
**III-R**	Autism	14	3.51	0.33	0.67
	Normal	20	3.46	0.19	
**V-R**	Autism	14	5.95	0.27	0.001
	Normal	20	5.55	0.29	
**I-III-R**	Autism	14	2	0.27	0.85
	Normal	20	1.96	0.24	
**III-V-R**	Autism	14	2.44	0.05	0.01
	Normal	20	2.09	4.05	
**I-V-R**	Autism	14	4.44	0.33	0.003
	Normal	20	4.05	0.37	
**I-L**	Autism	14	1.54	0.97	0.04
	Normal	20	1.49	0.08	
**III-L**	Autism	14	3.65	0.16	0.26
	Normal	20	3.57	0.39	
**V-L**	Autism	14	5.76	0.23	0.01
	Normal	20	5.42	0.32	
**I-III-L**	Autism	14	2.11	0.16	0.36
	Normal	20	2.08	0.39	
**III-V-L**	Autism	14	2.1	0.16	0.004
	Normal	20	1.85	0.42	
**I-V-L**	Autism	14	4.21	0.2	0.04
	Normal	20	3.93	0.38	
